# How do individuals decide whether to accept or decline an offer of genetic testing for colorectal cancer?

**DOI:** 10.1186/1897-4287-9-S1-P17

**Published:** 2011-03-10

**Authors:** Louise Keogh, Belinda McClaren, Judith Maskiell, Heather Niven, Alison Rutstein, Louisa Flander, Clara Gaff, John Hopper, Mark Jenkins

**Affiliations:** 1 Centre for Women’s Health, Gender and Society, The University of Melbourne, Melbourne, Victoria, 3010, Australia; 2 Centre for Molecular, Environmental, Genetic and Analytic Epidemiology, The University of Melbourne, Melbourne, Victoria, 3010, Australia; 3 Departments of Paediatrics and Medicine, The University of Melbourne, Melbourne, Victoria, 3010, Australia

## Background

Since 1997, the Victorian Colorectal Cancer Family Study (VCCFS) has been studying a large number of individuals who have had colorectal cancer and their relatives. When a genetic mutation is identified by the VCCFS, family members are offered the chance to have genetic testing through a family cancer clinic to learn of their result. Between 17% (before August 2003) and 49% (after August 2003) of individuals offered genetic testing decline the offer [1], providing a unique opportunity to conduct research with individuals who decline genetic testing. Understanding this decision making process is critical to improving the uptake of genetic testing in practice [2].

## Methods

A sample of participants in the VCCFS who had been offered genetic testing between 2003 and 2008 were invited to take part in a qualitative interview about genetic testing decision-making in the area of colorectal cancer. The sample included both those who declined genetic testing (decliners) and those who accepted genetic testing (acceptors). Thematic analysis was performed to determine the main reason individuals gave for accepting or declining the offer of genetic testing.

## Results

A total of 15 interviews have been conducted, six with decliners, and nine with acceptors. All participants recalled the offer of genetic information, and knew of the hereditary genetic mutation in their family. Each participant was able to clearly describe their decision. There were four types of decliners and four types of acceptors. Individuals declined because 1) Lynch Syndrome had been confirmed by other means; 2) fear about insurance implications; 3) genetic testing ‘wouldn’t change anything’ or 4) a positive result would cause too much worry. The main reason individuals accepted the offer of genetic testing was for the sake of 1) their children; 2) their own health; 3) curiosity; or 4) research. We consider how this decision was related to both risk perception and screening behaviour.

## Conclusions

The key perception informing the genetic testing decision was the consequence of receiving results. In the case of decliners, receiving genetic results was perceived to have either no consequences or negative consequences (financial or emotional). For acceptors, genetic testing was perceived to have positive consequences either for their children, themselves, or society. This research highlights the need to explore risk perceptions, genetic testing and screening decision making in more detail in families eligible for genetic testing, in order to increase the uptake of genetic testing in this group.
